# Paracentesis Thorasis

**Published:** 1857-09

**Authors:** 


*Paracentesis Thorasis.* — Dr. Bowdich warmly advocates a more fearless use of this operation, in ordinary cases of accumulation of fluid in the pleural cavity. His experience has certainly shown that the bug-bear of opening into the chest is one of those ideas which has been handed down to the teror of the surgeon, and delay of medical progress, until some bold man passes the Rubicon and proves the safety of an operation, hitherto avoided by practitioners.

Dr. Bowdich says, through the pages of the Boston Med. Journal, "since April 17, 1850, I have operated upon sixty-two individuals, of both sexes and all ages. I have punctured one hundred and eleven times. I know of nothing in practical medicine which has affo.ded me more satisfaction than this simple operation."

The name of Dr. Bowdich is a sufficient guarantee for the *honesty* of this statement; and a practitioner can hardly be said to give humanity the benifit of all that science can do if he suffers his patient to carry quarts of liquid in his chest, subject to the slow and uncertain action of absorption, when he can be relieved and the diagnosis made certain, by an operation which its distinguished advocate likens to venesection or vaccination. An operation so simple and so harmless, which procures such certain relief permanent or temporary, to a frequent and distressing malady, cannot be too strongly advocated in the periodical literature of the day. Let us make it another point in which we excell as *practitioners* our transatlantic brethren.

It seems probable that another important benefit may be derived from the early employment of thoracentesis. If it be performed before the lung has lost its elasticity by long compression, would it not expand more nearly to its former volume, and the contraction of the thoracic parietes, with the consequent unsightly dropping of the shoulder be lessened if not prevented? The value of this surmise requires extended observation for its establishment; but if it be true that the rapid and early evacuation of the fluid has any effect upon the deformity which attends recovery from thoracic effusion, it will add something to the value of an operation, the advantages of which must soon be felt in every day practice. F.

*Medical Department of the University of Louisville.*—This institution, during the last session, met with a serious calamity in the loss of the fine edifice appropriated to its use, together with the museum and a portion of the library and chemical apparatus. The building and its contents, however, were largely insured, and we are gratified to learn that a new structure will be in readiness for the approaching session. The new college, it is stated in the circular, will be greatly superior in every respect, to its predecessor, and, in fact, will have no superior in the United States. Extensive purchases have also been made, mainly in Europe, for a new museum, apparatus, etc., which it is expected will be received before the lectures commence.

Some changes in the faculty have recently occurred. Prof. Rogers has resigned the chair of theory and practice, and been elected professor of clinical medicine. Dr. Theodore S. Bell, of Louisville, has been elected to the chair vacated by Prof. Rogers. Prof. Bell was for many years an associate editor of the Western Med. & Surg. Journal, contributing largely to its pages. As a writer he is distinguished for varied knowledge, independence of thought, and a piquancy of style fitting him admirably for the post of an editor. He enters on the duties of a professor at a mature age, with a large experience as a practitioner, and with habits of application (heretofore directed to a multiplicity of subjects) such as are rarely found even by the most industrious of the members of the medical profession. Notwithstanding the cares and disastions incident to practice, few men in our country have given to daily study so many hours.

We cordially wish for this institution continued prosperity. *

---

*Excito-Secretory System of Nerves. Dr. H. F. Campbell.*—The priority of the idea of a portion of the nervous system belonging specially to secretion, appears to be conceded on all hands to our countryman, Dr. H. F. Campbell, of Augusta, Georgia. Dr. Campbell's first publication relative to this subject, was in 1850. He enunciated the idea more distinctly in a report made to the American Medical Association in 1853, and again more recently in an essay which gained the prize at the last meeting of the association. Marshall Hall, who had been led to recognize in the functions of the nervous system this new division, resigns the merit of the discovery in favor of Dr. Campbell.

Now that the question of priority is settled, it remains to render unquestionable the validity of the discovery itself. The experimental researches of Bernard go far toward establishing the existence of an excito-secretory system. We may present, hereafter, to our readers, facts relating to the subject.

Dr. Campbell has recently received the appointment of professor of anatomy in the medical college of Georgia. He has for several years been connected with that institution as demonstrator of anatomy. He succeeds, in the chair of anatomy, Dr. George M. Newton, resigned. Dr. Newton has enjoyed the reputation of being one of the best teachers of anatomy in the country. *

---

*New Orleans School of Medicine.*—Dr. A. Foster Axon, the professor of physiology, has been compelled by the state of his health and the pressure of other occupations, to resign his chair in this institution. Dr. Anthony Perriston, the adjunct professor of anatomy, has been appointed to the vacancy; and Dr. Theodore S. Clapp will fill the vacancy made by the appointment of Dr. Perriston.

By reference to the circular it will be seen that the faculty propose, "to give experimental, chemical, and microscopic physiology a prominent position in the curriculum of studies." Dr. Perriston has already spent three years in Europe in the pursuit of his favorite studies. He will spend the following summer, however, in Paris, and will make valuable additions to the museum and library of the institution.

We are glad to see a disposition in medical colleges to encourage the application of the microscope and vivisection to the teaching of physiology. Experimental and demonstrative physiology was first taught in this country, at the Buffalo Medical College, in 1851–2, by Prof. John C. Dalton, Jr., who repeated before the class, the brilliant demonstrations of M. Bernard; since then, the example has been followed by many of our teachers of this branch, and we trust that so interesting and impressive a mode of instruction may be more generally adopted. F.

---

*Fusion of the Medical Schools in Cincinnati.*—The two rival medical schools at Cincinnati, have amalgamated, under the title of the Medical College of Ohio, the name of the older of these institutions. This union involves some changes in the corps of faculties. Professor S. G. Armor has resigned his chair in the Ohio Medical College, and has accepted, as we learn, a professorship in St. Louis. Professors Marshall, Worder, and Tate, also, connected formerly with that college, have withdrawn. The venerable Professor Mussey, of late connected with the Miami school, retires. Professors Judkins, Comegys, Foote, and Mendenhall, formerly of the last named school, remains in the present organisation. Inasmuch as two medical schools are not needed in Cincinnati, and the immediate proximity of rival institutions to each other is apt to lead to evils affecting the prosperity of both, as well as impairing the comfort of those connected therewith, this consolidation must be (to use a quotation occasionally employed by writers) a "consummation devoutly to be wished." *

---

*Rush Medical College.*—Some changes have taken place in this institution since the close of the last college term. Professors Evans and Herrich have resigned, and their places have been filled as follows: W. H. Byford, M. D., chair of obstetrics; John H. Ranch, M. D., chair of materia medica, etc. Prof. Johnson, formerly the incumbent of the chair last named, has been transferred to the chair of physiology and pathology, made vacant by the resignation of Prof. Herrich. The faculty of the college passed complimentary resolutions on the occasion of the retirement of Professors Evans and Herrich. *

---

*Jefferson Medical College.*—Dr. Thomas D. Mitchell has been elected to the chair of Materia Medica and Therapeutics in the Jefferson Medical College, in place of Dr. Huston, who has recently resigned in consequence of ill-health. Dr. Mitchell is favorably known as the author of a large and practical work on therapeutics. He has long been a teacher of this branch of medical science, having formerly been for many years connected with the Transylvania Medical College, at Lexington, Ky., and, also, with the Medical College of Ohio. *

---

*Trouble among the Wayne Co. Doctors.*—The Wayne County Medical Society held a meeting the other day and appointed a committee of three to investigate the following charges against one of its members, viz: "For violating a rule of the society and of medical ethics, by advertising publicly and by handbills, inviting the attention of individuals affected with particular diseases of the lungs, &c., professing superior skill and knowledge, these being the ordinary practices of empirics, &c," The charges were sustained and *the member expelled!*

---

Her Majesty Queen Victoria, has been graciously pleased to bestow the honor of knighthood on her chief accoucheur, Dr. Locock. It has been suggestested, however, that this distinguished gentleman deserved, after his arduous duties, to have been elevated to the peerage under the title of Earl Deliver us!—*Montreal Monthly Jour. of Med. & Surg.*

The above item of medical news had a place in our number for June, with the slight difference, that the title there quoted as most appropriate for Sir Charles Locock, was "Lord Deliver us," instead of "Earl Deliver us," as our Canadian friend has it. Perhaps he does not see the point, but we can assure him that "it created a great laugh at the time." F.

